# Drug Poisonings among Patients Presenting to the Emergency Department of a Tertiary Care Center in Eastern Nepal: An Observational Study

**DOI:** 10.31729/jnma.v63i2091.9240

**Published:** 2025-11-30

**Authors:** Namita Kumari Mandal, Rabin Bhandari, Gajendra Prasad Rauniyar, Madan Krishna Mandal

**Affiliations:** 1 Department of Clinical Pharmacology and Therapeutics, B.P. Koirala Institute of Health Sciences, Dharan, Sunsari, Nepal; 2 Department of General Practices and Emergency Medicine, B.P. Koirala Institute of Health Sciences, Dharan, Sunsari, Nepal; 3 Department of Emergency Medicine, Nobel Medical College Teaching Hospital, Biratnagar, Morang, Nepal

**Keywords:** *drug poisoning*, *benzodiazepines*, *analgesics*, *intentional self-poisoning*

## Abstract

**Introduction:**

Drug poisoning is a major public health concern contributing to emergency visits and hospital admissions. Understanding the patterns and characteristics of drug poisoning cases is crucial for developing targeted prevention strategies, improving early recognition, guiding appropriate clinical management, and informing public health policies to reduce morbidity and mortality. This study aimed to determine the prevalence and pattern of drug poisoning cases presenting to the Emergency Department of a tertiary care center in Eastern Nepal.

**Methods:**

A prospective observational study was conducted in the Emergency Department of a tertiary care hospital from 1 January to 31 December 2022, after ethical approval (Ref. No: IRC/1466/018). Data from 60 eligible patients were collected using a structured proforma including socio-demographics, type and route of drug exposure, intent (accidental or suicidal), time of presentation, clinical features, management, and short-term outcomes. Descriptive statistics were used for analysis.

**Results:**

Among 30,019 emergency admissions, 60 (0.20%) were due to drug poisoning, with 38 (63.33%) cases accounting for females. Intentional self-poisoning occurred in 59 (98.33%) cases, while 1 (1.67%) case was accidental. Among the reported cases, 21 (35.00%) cases involved the use of CNS depressants (benzodiazepines), 18 (30.00%) cases involved analgesics, and 2 (3.33%) cases involved antidepressant use. There were 13 (21.76%) patients who presented within six hours of ingestion and received timely supportive care. There was 1 (1.67%) case of mortality.

**Conclusions:**

Intentional self-poisoning was the predominant form of drug poisoning, mainly involving benzodiazepines and analgesics among young females. Most patients recovered with supportive management, and the case fatality rate was low.

## INTRODUCTION

Drug overdose is a significant public health issue globally and in Nepal, contributing to both intentional and unintentional poisonings.^1^ The Global Burden of Disease 2019 reports the age-standardized death rate for unintentional poisoning in South Asia of 0.41 per 100,000, with substantial disability-adjusted life years (DALYs).^2^ In Nepal, hospital-based studies report a notable number of emergency admissions due to drug overdose, highlighting its clinical importance.^3-6^ Intentional poisonings involve suicide attempts, while unintentional poisonings result from dosing errors or accidental ingestion. Easy access to medications, limited regulation, rising mental health issues, and post-COVID-19 challenges may increase incidence, especially in young adults and females.^1,3-7^

Commonly implicated drugs include benzodiazepines, antidepressants and over-the-counter analgesics.^3-6,8^ Understanding these patterns is essential to guide preventive strategies, improve emergency care, and inform health care planning.

This study describes the prevalence, socio-demographics, drug types, clinical features, management, and short-term outcomes of drug poisoning cases in a tertiary care emergency department in Eastern Nepal.

## METHODS

This descriptive prospective observational study included all patients presenting with drug poisoning to the Emergency Department (ED) of a tertiary care center in Eastern Nepal from 1st January 2022 to 31st December 2022. Ethical approval was obtained from the Institutional Review Committee (IRC) of BP Koirala Institute of Health Sciences (IRC/1466/018). Due to COVID-19 safety restrictions, participant recruitment was initially postponed. Data collection commenced following confirmation from the ethics committee that the original approval remained valid. Written informed consent was obtained from participants or guardians in cases where patients were unconscious.

Drug poisoning was defined according to the World Health Organization ICD-10 classification as the harmful ingestion of pharmaceutical agents leading to clinical symptoms or hospitalization.^9^ Data on demographic characteristics, type and dose of the ingested drug, time and mode of poisoning, and short-term clinical outcomes were collected using a structured proforma. To minimize recall bias, data were collected once patients were clinically stable and cross-verified with attendants, medication strips, referral documents, prescriptions, or prior medical records. For unconscious patients, reliable witnesses such as family members, caregivers, or referring staff were interviewed. Although toxicological confirmation was not performed, this process allowed approximate validation of drug identity and dose. The identity of the ingested drug could not be confirmed in 16 (26.67%) cases, which may limit the interpretation of drug-specific patterns and outcomes.

Individuals aged 15 years or older presenting to the emergency department with drug poisoning during the study period and able to provide informed consent were included (the total sampling method was used). During the study period, 65 patients presented with suspected poisoning, of which 5 were excluded: 3 due to nondrug substances (household chemicals) and 2 due to incomplete data. Exclusion criteria comprised poisoning from non-drug substances (such as pesticides, household chemicals, industrial agents, natural toxins, and alcohol). Cases with incomplete data, unclear history of the substance ingested, patients brought dead, or those with missing key variables, including incomplete triage or unavailable short-term outcome data, were also excluded, which may introduce selection bias. The final sample included 60 patients, reflecting the total number of eligible cases recruited during the study period. To ensure complete case capture in this prospective study, the Emergency Department’s 24-hour patient log was reviewed daily by the investigators. Patient severity at presentation was categorized using the Australasian Triage Scale (ATS), a five-level system (ATS 1: Immediate resuscitation - life-threatening, needs treatment within 0 minutes, ATS 2: Emergency - high risk, should be seen within 10 minutes, ATS 3: Urgent - should be seen within 30 minutes, ATS 4: Semiurgent - should be seen within 60 minutes, ATS 5: Non-urgent - can safely wait up to 120 minutes).^10^

All patients with intentional self-poisoning were referred for psychiatric assessment during their hospital stay. Psychiatric evaluation was performed by a qualified psychiatrist or psychiatry resident according to hospital protocol to assess mental health status, risk of recurrence, and need for follow-up.

Data were analyzed using the Statistical Package for the IBM SPSS Statistics for Windows, version 11.5. Only descriptive statistics were used: continuous variables are presented as mean ± standard deviation, and categorical variables as frequency and percentage. Results are presented in tabular form.

## RESULTS

Of the 30,019 poisoning patients presenting to the Emergency Department during the study period, 60 (0.20%) had drug poisoning. Among the 60 patients included in the study, the gender distribution was a female-to-male ratio of 1.7:1. The median age was 24 years (IQR: 19-28), with 45 (75.00%) patients aged 15-30 years. There were 27 (45.00%) students and 19 (31.67%) homemakers among occupational groups. There were 46 (76.67%) poisoning incidents that occurred at home and 38 (63.33%) during nighttime hours. The oral route was the most common method of drug ingestion. There were 45 (75.00%) patients who presented to the Emergency Department within 4 hours of ingestion. Intentional poisoning accounted for 59 cases (98.33%). There were 50 (83.33%) patients who arrived at the emergency department with minimal pre-hospital care. Patients from Sunsari district accounted for 35 (58.33%) of the cases (Table 1).

**Table 1:** Socio-demographic findings of patients with drug poisoning cases (n=60).

Category/Variable	Number of cases n(%)
**Gender**	
Male	22(36.67)
Female	38(63.33)
**Age group (years)**	
15-30	45(75.00)
30-45	12(20.00)
45-60	3(5.00)
>60	0(0)
**Marital Status**	
Unmarried	26(43.33)
Married	34(56.67)
**Educational status**	
Literate	59(98.33)
Illiterate	1(1.67)
**Occupation**	
Students	27(45.00)
Homemakers	19(31.67)
Manual worker	9(15.00)
Farmer	2(3.33)
Shopkeeper	2(3.33)
Health care professional	1(1.67)
**Address**	
Sunsari	35(58.33)
Morang	10(16.67)
Jhapa	6(10.00)
Outside Koshi province	9(15.00)

Central nervous system depressants such as benzodiazepines were implicated in 21(35.00%) patients, followed by paracetamol in 18 (30.00%) patients. Antidepressants and thyroxine were used in 2 (3.33%) cases each, while the offending agent was unknown in 16 (26.67%). N-acetylcysteine, an antidote, was administered only to patients with suspected significant paracetamol ingestion. In the remaining cases, where the ingested dose appeared minimal and laboratory parameters, including liver and renal function tests, were within normal limits, supportive care alone was provided. Toxicological screening was not available.

Neurological symptoms were seen in 27 (45.00%) patients, presenting as sleepiness in 22 (36.67%) cases, unresponsiveness in 3 (5.00%) cases, and disorientation in 2 (3.33%) cases. Gastrointestinal manifestations occurred in 20 (33.33%) patients, including abdominal pain in 15 (25.00%) cases, nausea in 3 (5.00%) cases, and vomiting in 2 (3.33%) cases. There were 13 (21.67%) cases that were asymptomatic at presentation. There were 55 (91.67%) patients who were alert, while 5 (8.33%) had altered sensorium (Table 2).

**Table 2:** Characteristics of drug poisoning cases by type of poison, clinical presentation, and outcome (n= 60).

Category/Variable	Number of cases n(%)
**Circumstances of poisoning**	
Suicidal	59(98.33)
Accidental	1(1.67)
**Type of Poison**	
CNS depressants	21(35.00)
Paracetamol	18(30.00)
Thyroxine	2(3.33)
Antidepressants	2(3.33)
Lindane	1(1.67)
Unknown	16(26.67)
**Emergency admissions**	
Direct	52(86.67)
Referred cases	8(13.33)
**Time of hospital arrival after poison in-take (hours)**	
≤ 4	45(75.00)
4-8	13(21.67)
>8	2(3.33)
**Disability Scale**	
Alert	55(91.67)
Response to Verbal Stimuli	2(3.33)
Response to Painful Stimuli	0
Unresponsive	3(5.00)
Response to verbal stimuli	2 (3.33)
Response to painful stimuli	0 (0.00)
Unresponsive	3 (5.00)

On arrival, 56 (93.3%) patients were categorized as Australasian Triage Scale (ATS) 2, while 4 (6.67%) patients were categorized as ATS 3, representing urgent cases to be seen within 30 minutes. Hypoglycemia was documented in 4 (6.67%) patients. Mean vital parameters were within the normal range (Table 3).

Among the 60 patients, 45 (75.00%) patients recovered following supportive management, which included monitoring, intravenous fluids, symptomatic care, antidotes if any, and psychiatric referral. They were discharged from the ED or transferred to the medicine ward after stabilization. There were 2(3.33%) patients who required intubation, among whom 1 (1.67%) patient had CNS depressant (benzodiazepine) ingestion and 1 (1.67%) patient had ingested an unknown agent. There was 1 (1.67%) patient with benzodiazepine poisoning who presented approximately 6 hours after ingestion. Despite intensive supportive care, including airway management and monitoring in the ED, the patient expired. There were 12 (20.00%) patients who were discharged against medical advice, 1 (1.67%) was discharged on request by the patient party, and 1 (1.67%) was referred because ICU facilities were unavailable during emergency admission, all after stabilization of vital signs (Table 4).

**Table 3:** Triage vitals at the time of arrival.

Triage vital parameters	Mean ± Standard Deviation)
Heart Rate	86.6±13.60
Respiratory Rate	21.2±1.90
Body Temperature (° F)	98±0.56
Systolic Blood Pressure (SBP)	116.8 ± 20.20
Diastolic Blood Pressure (DBP)	73±14
Oxygen Saturation (%)	97.5±1.90

**Table 4:** Emergency management and outcomes of patients with drug poisoning (n=60).

Parameters	Number of cases n (%)
Requirement of Intubation	
Yes	2 (3.33)
No	58 (96.67)
Emergency Outcome of patients	
Shifted to Medicine ward or Discharged from Emergency	45 (75.00)
Discharged Against Medical Advice (DA-MA)	12 (20.00)
Discharge on Request (DOR)	1 (1.67)
Refer	1 (1.67)
Death	1 (1.67)

## DISCUSSION

This study describes the demographic and clinical characteristics of patients presenting with drug poisoning to the Emergency Department of a tertiary care center in Eastern Nepal. During the one-year study period, a total of 30,019 patients attended the ED, among whom 60 cases were due to drug poisoning. This corresponds to a prevalence of 0.20% (95% CI: 0.15-0.25%). The most 45 (75%) patients were young adults (15-30 years), and females were affected more frequently. The prevalence at our center falls within the global range reported by Okumura et al. and Xiang et al.(17-237 per 100,000), indicating that drug poisoning represents a measurable but relatively uncommon cause of ED visits in this setting. ^11,12^

Reported prevalence of drug poisoning varies widely across studies. In Nepal, hospital-based studies have reported that drug poisoning accounts for 7.29% to 22.77% of emergency department visits reflecting local healthcare access and reporting patterns. ^5,13^ In neighbouring country India, a hospital-based study reported a prevalence of 27.6%, while international studies have documented rates as low as 4.6% in Ellison et al. ^6,14^ These differences likely reflect variations in study populations, health care infrastructure, referral patterns, and case definitions. The relatively low prevalence observed at our center may be influenced by underreporting of minor poisonings, exclusion of pre-hospital deaths, and referral practices typical of a tertiary care center. Most cases in our study involved young adults and females, consistent with demographic patterns reported in regional studies.^5,6,13^ Commonly implicated drugs included Central Nervous System (CNS) depressants like benzodiazepines and over-the-counter analgesics, highlighting the importance of regulating medication access, improving public awareness, and integrating mental health support into preventive strategies.^6,13,14^

In this study, 45(75%) of drug poisoning cases occurred in individuals aged 15-30 years, consistent with reports from Nepal and neighbouring South Asian countries showing peak incidence among young adults.^5,6,13,14^'^26^ This age group may be particularly vulnerable due to psychosocial stressors such as academic pressure, relationship issues, unemployment, and emerging mental health challenges. Similar patterns have been observed in regional studies highlighting the role of easy access to medications and sociocultural factors.^7,19-21^ However, some studies from other regions have reported higher poisoning rates in children, likely reflecting differences in demographic profiles, healthcare access, household safety practices, and awareness levels.^18,20-22^

The predominance of young adults in our findings has important clinical and public health implications. Clinicians should be particularly vigilant when treating patients in this age group, as many cases may involve intentional self-harm or suicide attempts rather than accidental ingestion.^6,16,17,19,23^ This pattern reflects the broader psychosocial context in which young individuals may face significant psychological stress due to academic pressure, interpersonal conflicts, unemployment, or emerging mental health problems.^5,6,13,14,20-26^ The post-pandemic period has been marked by increased psychosocial stress and emotional instability among young individuals, which may have influenced the pattern of intentional poisonings.^27,28^ Addressing these underlying causes through integrated psychiatric assessment, counselling, and community-based mental health awareness programs could help mitigate such events. Moreover, public health initiatives should focus on mental health education, life skills training, and emotional support targeting adolescents and young adults. Community-based awareness programs and school- or college-level interventions could help reduce the incidence of self-poisoning.^6,7^ Early identification of at-risk individuals through primary care screening and referral systems can also be instrumental in prevention.

Most poisonings occurred at home at night, and a high proportion of patients were young adults and females, consistent with regional reports.^6,14-21^ In our study, 45(75%) presented within 4 hours, and 59(98.3%) were intentional poisonings—similar to prior studies.^11,14-17,23^ Most arrived directly at the emergency department, with minimal pre-hospital care. The predominance of intentional poisoning and the short interval to hospital presentation indicate opportunities for early intervention. Minimal pre-hospital care highlights gaps in emergency preparedness and referral systems. These findings underscore the need for mental health awareness programs, suicide prevention strategies, and strengthened emergency response, particularly in academic and community settings.

In our study, the most frequently implicated agents were CNS depressants such as benzodiazepines in 21(35.00%), followed by paracetamol/analgesics in 18(30.00%) and antidepressants in 2(3.33%) cases, consistent with findings by Sanjay M et al. and Okumura et al.^6,11^ While partially aligning with other studies, variations were noted—Vanishree B et al. reported paracetamol as the leading agent, Zaki et al. highlighted sedatives and opioids, Bell et al. and Dal et al. emphasized antidepressants, and Mbaroak et al. observed mixed drug use.^15,18,19,23,26^ Most of the drugs involved had been previously prescribed to patients or were readily available at home, indicating inappropriate storage and easy accessibility. A smaller number were reportedly obtained without prescription from local pharmacies. Clinical records and history suggested that many cases, particularly among young adults, were associated with emotional distress or possible underlying mental health issues. These findings emphasize the need for stricter regulation of prescription drug dispensing and improved mental health screening at the community level. The ‘unknown’ drug category accounted for 16(26.67%) cases. Patients in the ‘Unknown’ drug category did not show significantly different short-term outcomes compared to known drug categories like benzodiazepines and paracetamol. Only one patient died during the emergency stay from this group, but the lack of drug-specific data limits interpretation of severity and management.

Clinicians should be vigilant when prescribing these medications and educate patients about their risks. Additionally, unknown drug ingestion in over a quarter of cases highlights the need for improved toxicological screening capacity and public education on safe drug storage and disposal.

Fifty six (93.33%) patients presented with stable vital signs, defined as heart rate 60-100/min, blood pressure 90-140/6090 mmHg, and respiratory rate 12-20/min, consistent with findings from Zaki et al., Reda et al., and Mbaroak et al.^18,20,23^ Random blood sugar (RBS) was within normal limits in 54(90%) cases, similar to reports by Zaki et al. and Mbaroak et al..^18,23^ Neurological symptoms were the most frequent and seen in 27(45.00%) patients, followed by gastrointestinal manifestations in 21(33.33%) cases, aligning with prior studies.^20,21,24,26^ Antidotes were administered in 13(21.67%) patients, primarily for paracetamol overdose, which is higher than Dal et al.(8.7%) and Reda et al.(12.8%), but lower than Vanishree B et al.(45.9%).^15,20,26^

The predominance of stable vital signs and normal random blood sugar levels at presentation, despite frequent neurological and gastrointestinal manifestations, suggests that apparent clinical stability may mask underlying toxicity. Subtle symptoms such as sleepiness or mild abdominal discomfort highlight the need for careful symptom evaluation and detailed toxicological history in all suspected cases. Antidotes were administered mainly for paracetamol overdose, emphasizing the importance of early identification of the offending drug and prompt initiation of specific therapy. Strengthening emergency preparedness through the availability of essential antidotes, timely psychiatric assessment for intentional cases and regular staff training in poisoning management protocols can further strengthen emergency preparedness and improve clinical outcomes in drug poisoning cases.

The mortality rate was low (1 case, 1.67%), notably lower than rates reported by Saravani et al. and Schwake et al. (16-17.3%).^17,25^ Forty five patients (75%) recovered fully. The median time from ingestion to ED presentation was 3 hours (IQR: 2-5 hours), indicating that early arrival may have contributed to favourable outcomes. These results are consistent with findings by Zaki et al., Bell et al., and Mbaroak et al., who also reported high recovery rates.^18,19,24^ Two patients (3.33%) required intubation— one following ingestion of a CNS depressant and the other due to an unknown agent. One patient (1.7%) with benzodiazepine poisoning died despite intensive management.

In this study, 14(23.33%) patients were discharged on request, left against medical advice, or were referred due to limited ICU availability. Their recovery status at discharge was not consistently documented, reflecting a common challenge in poisoning management. Similar findings were reported by Bell et al., who observed a 9% DAMA rate, and Pavarin et al., who noted mortality ranging from 13.7 (females) to 16.6 (males) per 1000.^19,29^ In the Nepalese context, factors such as mental health stigma, limited awareness of psychiatric services, financial constraints, and family influence may contribute to early discharge.^30^ Such cases may not receive complete medical stabilization or psychological assessment, increasing the likelihood of recurrence or poor long-term outcomes.

Our findings are consistent with national and international studies regarding prevalence, age, gender, and commonly implicated drugs, although variations likely reflect differences in local drug access and healthcare-seeking behaviours.^5,6,11-26^ These results highlight the need for standardized poisoning protocols, adequate antidote availability, and staff training. Preventive efforts should focus on public education, safe medication disposal, and integration of mental health care into primary services. This study has several limitations. First, it was conducted at a single tertiary care center, which may limit generalizability. Second, the sample size was relatively small (n=60), and drug identification relied on patient or attendant recall without comprehensive toxicological confirmation, potentially introducing misclassification bias. Third, prehospital deaths were not included, and 14(23.3%) patients were discharged against medical advice (DAMA), which may lead to underestimation of complications and mortality. Finally, as a tertiary care center, our hospital may preferentially receive sicker or referred cases, introducing referral bias. Despite these limitations, a key strength of the study is the systematic documentation of drug poisoning cases in a real-world, resource-limited emergency setting. Future research should consider multi-center surveillance, incorporation of laboratory toxicology, and longitudinal follow-up to assess psychiatric outcomes and long-term prognosis of poisoning cases.

The single-centre, cross-sectional design limits generalizability and prevents causal inference. Despite these limitations, the study provides important evidence in a setting with scarce data on drug poisoning and can guide future multi-centre or community-based research.

## CONCLUSION

Drug poisoning in this single-centre study predominantly affected young adults, particularly females, through intentional ingestion of commonly available medications. Most patients recovered with supportive care, although a notable proportion left against medical advice or on request. These findings highlight the demographic and clinical patterns of drug poisoning in a tertiary care emergency setting and underscore the importance of timely assessment, close monitoring, and integration of mental health support in patient management.

## Data Availability

The data are available from the corresponding author upon reasonable request Ethical Considerations: Ethical issues (including plagiarism, data fabrication, and double publication) have been completely observed by the authors.

## References

[1] World Health Organization (2022). Global Health Estimates 2022.. https://www.who.int/data/global-health-estimates.

[2] Global Burden of Disease Collaborative Network (2020). Global Burden of Disease Study 2019 (GBD 2019).. https://ghdx.healthdata.org/gbd-2019.

[3] Nepal S, Atreya A, Regmi P, Shrestha P, Shrestha RB, Sapkota LP (2025). Epidemiology of Poisoning Cases at Lumbini Medical College, Nepal: A 5-Year Retrospective Study.. Health Sci Rep.

[4] Thakali K, Ulak N, Bharati M, Thapa LJ, Paudyal DN, Basnet CK (2022). Poisoning Among Patients Presenting to the Department of Emergency Medicine of a Tertiary Care Centre: A Descriptive Cross-Sectional Study.. J Nepal Med Assoc.

[5] Bhandari R, Bhandari R, Gupta PP (2017). Trend and Outcome of Acute Poisoning Case: An Experience From Emergency Department of Eastern Nepal.. Int J Community Med Public Health.

[6] Sanjay M, Rabbi Abel, Jain S, Abhilash A, Kundavaram PP (2023). Profile of Patients Presenting With Deliberate Drug Overdose and Outcome.. Med J Dr DY Patil Vidyapeeth.

[7] Thapaliya S, Sharma P, Upadhyaya K (2018). Suicide and Self Harm in Nepal: A Scoping Review.. Asian J Psychiatr.

[8] Kamal M, Negm WA, Abdelkader AM, Alshehri AA, El-Saber Batiha G, Osama H (2023). Most Common Over-the-Counter Medications and Effects on Patients.. Eur Rev Med Pharmacol Sci.

[9] World Health Organization (2016). International Statistical Classification of Diseases and Related Health Problems, 10th Revision (ICD-10).. https://icd.who.int/browse10/2016/en.

[10] Australasian College for Emergency Medicine (2013). Australasian Triage Scale.. https://acem.org.au/Content-Sources/Advancing-Emergency-Medicine/Better-Outcomes-for-Patients/Triage.

[11] Okumura Y, Sakata N, Takahashi K, Nishi D, Tachimori H (2017). Epidemiology of Overdose Episodes From the Period Prior to Hospitalization for Drug Poisoning Until Discharge in Japan: An Exploratory Descriptive Study Using a Nationwide Claims Database.. J Epidemiol.

[12] Xiang Y, Zhao W, Xiang H, Smith GA (2012). ED Visits for Drug-Related Poisoning in the United States, 2007.. Am J Emerg Med.

[13] Kishore P, Palaian S, Paudel R, Mishra D, Ojha P, Alam K (2008). Pattern of Poisoning Cases in a Teaching Hospital in Western Nepal.. J Int Med Nepal.

[14] Ellison J, Walley AY, Feldman JA, Bernstein E, Mitchell PM, Koppelman EA (2016). Identifying Patients for Overdose Prevention With ICD-9 Classification in the Emergency Department, Massachusetts, 2013-2014.. Public Health Rep.

[15] Vanishree B, Begum A (2022). Management and Outcome of Different Drug Overdose Cases in a Tertiary Care Hospital - A Prospective Observational Study.. Natl J Physiol Pharm Pharmacol.

[16] Al-Jahdali H, Al-Johani A, Al-Hakawi A, Arabi Y, Ahmed QA, Altowirky J (2004). Pattern and Risk Factors for Intentional Drug Overdose in Saudi Arabia.. Can J Psychiatry.

[17] Saravani K, Afshari M, Aminisefat A, Bameri O (2021). Blood Sugar Changes in Patients With Acute Drug Poisoning.. Cell Mol Biomed Rep.

[18] Zaki AR, Ghaleb SS, Abdelmenem A, Yousef MA (2019). Retrospective Study of Addictive Drug-Induced Acute Toxicity of Cases Admitted to the Poison Control Centre of Ain Shams University Hospital (2015-2016).. Egypt J Forensic Sci.

[19] Bell M, Holbrook A, Wallace C, Hanel E, Rigg K (2024). Characteristics of Drug Poisonings Seen in the Emergency Department of an Urban Hospital.. Can J Hosp Pharm.

[20] Reda W, Bakhashwain NA, Albehiri SA, Alghamdi HA, Jumah AA, Eibani WK (2023). Drug Overdose Patterns Among Emergency Department Patients at an Academic Hospital in Jeddah.. Cureus.

[21] Abdel Rasheed EAM, Elmahdy N, Shalaby E, Karoube HS, Badawy SM (2020). A Study of Pharmaceutical Drugs Poisoning Cases Admitted to the National Poisoning Center, Kasralainy Teaching Hospital in Cairo.. Eurasian J Emerg Med.

[22] Bakhaidar M, Jan S, Farahat F, Attar A, Alsaywid B, Abuznadah W (2015). Pattern of Drug Overdose and Chemical Poisoning Among Patients Attending an Emergency Department, Western Saudi.. J Community Health.

[23] Mbarouk GS, Sawe HR, Mfinanga JA, Stein J, Levin S, Mwafongo V (2017). Patients with Acute Poisoning Presenting to an Urban Emergency Department of a Tertiary Hospital in Tanzania.. BMC Res Notes.

[24] Jones CM, Mack KA, Paulozzi LJ (2013). Pharmaceutical Overdose Deaths, United States, 2010.. JAMA.

[25] Schwake L, Wollenschläger I, Stremmel W, Encke J (2009). Adverse Drug Reactions and Deliberate Self-Poisoning as Cause of Admission to the Intensive Care Unit: A 1-Year Prospective Observational Cohort Study.. Intensive Care Med.

[26] Dal E, Eraybar S (2024). Retrospective Analysis of Poisoning Cases Presenting to the Emergency Department After Drug Intake: Demographic Characteristics and Necessity of Antidote Use.. Glob Emerg Crit Care.

[27] Sharma P, Bhandari AR, Shakya R, Sapkota N, Joshi S, Bhattarai G (2024). Development and Use of Suicide Registry for Recording Patient Profile With Self-Harm Visiting Tertiary Hospital of Nepal: A Descriptive Cross-Sectional Study.. JNMA J Nepal Med Assoc.

[28] Banerjee I, Robinson J, Sathian B, Banerjee I (2021). COVID-19 Pandemic and Suicides in Nepal: Way Forward for Prevention.. Nepal J Epidemiol.

[29] Pavarin RM, Berardi D, Gambini D (2016). Emergency Department Presentation and Mortality Rate Due to Overdose: A Retrospective Cohort Study on Nonfatal Overdoses.. Subst Abus.

[30] Sharma GRP, Adhikari SK, Raibanshi L (2025). Reasons for Patients Leaving Against Medical Advice in Emergency Department at a Tertiary Hospital, Nepal.. JGMC-Nepal.

